# Dope-Dyeing of Polyvinyl Alcohol (PVA) Nanofibres with Remazol Yellow FG

**DOI:** 10.3390/polym12123043

**Published:** 2020-12-18

**Authors:** Fatirah Fadil, Farah Atiqah Adli, Nor Dalila Nor Affandi, Ahmad Mukifza Harun, Mohammad Khursheed Alam

**Affiliations:** 1 Textile Research Group, Faculty of Applied Sciences, Universiti Teknologi MARA, Shah Alam 40450, Selangor, Malaysia; fatirahfadil@uitm.edu.my (F.F.); farahatiqahadli@gmail.com (F.A.A.); 2 Engineering Faculty, Universiti Malaysia Sabah, Jalan UMS, Kota Kinabalu 88400, Sabah, Malaysia; mukifza@ums.edu.my; 3 College of Dentistry, Jouf University, Sakaka 72721, Saudi Arabia; dralam@gmail.com

**Keywords:** dope-dyeing, dye content, electrospinning, polyvinyl alcohol, nanofibres morphology

## Abstract

The lack of aesthetic properties of electrospun nanofibres in terms of colour appearance is the drive in this preliminary study. This research is conducted to study the dyeing behaviour and colorimetric properties of electrospun nanofibres blended with Remazol Yellow FG reactive dye using dope-dyeing method via electrospinning process. This paper reports the colorimetric properties of dyed poly vinyl alcohol (PVA) nanofibres within the range of 2.5 wt.% to 12.5 wt.% dye content. The electrospinning parameters were fixed at the electrospinning distance of 10 cm, constant feed rate of 0.5 mL/h and applied voltage of 15 kV. The resulting impregnated dye of 10 wt.% exhibits acceptable colour difference of dyed PVA nanofibres, with a mean fibre diameter of 177.1 ± 11.5 nm. The SEM micrographs show the effect of dye content on morphology and fibre diameter upon the increment of dye used. Further increase of dye content adversely affects the jet stability during the electrospinning, resulting in macroscopic dropping phenomenon. The presence of all prominent peaks of Remazol dye in the PVA nanofibers was supported with FTIR analysis. The addition of dye into the nanofibres has resulted in the enhancement of thermal stability of the PVA as demonstrated by TGA analysis.

## 1. Introduction

In principle, nanomaterials can be defined as any material having particles or constituent of nanoscale dimensions. The realm of nanomaterials involves an extensive range of fascinating materials with exceptional physical and chemical properties and characteristics [1]. These nanomaterials include zero-dimensional (0D) nanoparticles, one-dimensional (1D) nanowires, nanotubes and nanofibres and two-dimensional (2D) nanosheets, which are manufactured through nanotechnology and used at a very small scale [2,3]. Among these nanomaterials, 1D nanostructured fibres prepared by electrospinning processes are at the forefront of scientific research driven by their extraordinary properties [4,5]. One of the prominent features of nanofibres is their exceptionally high surface area-to-volume ratio and high porosity or tortuosity. These features of materials are most desirable for advanced application including protective clothing, functional textiles, chemical and biological sensors and tissue engineering [6,7]. Hence, researchers now have recognized that electrospun nanofibres has great potential in the textiles industries, as such these fibres have compromised the good comfort, thermal insulation and good permeability to air properties [8]. The huge potential of electrospun nanofibres in textiles applications is exhibited through its excellent durability and free from deteriorating effect prior to laundering [9]. A previous study led by Yoon and Lee (2010) has highlighted the enhanced waterproofing and breathability properties of electrospun nanofibres in comparison to the conventional non-woven textile [10].

Although electrospun nanofibres had many promising advantages over conventional textile, the application of electrospun nanofibres in textile industries is comparatively novel and is limited due to several factors such as colour limitation of as-spun nanofibres. Electrospun nanofibres mat is commonly seen in white, due to the light scattering phenomenon [11]. As for the interaction of light with nanomaterials, a single nanofibre is not visible to the naked eye or through an optical microscope due to its nanoscale diameter, as it is in the same range of magnitude with the visible light wavelengths. Conversely, as the layer of nanofibres becomes thicker, the surface of nanofibers mat will be illuminated by white light, and consequently it will appear in white colour, opaque and smooth surface. This light scattering phenomenon is known as light-nanofibres interaction [12]. In order to overcome the limitation of light-nanofibres interaction, the idea of dope-dyeing of electrospun nanofibres via electrospinning is proposed in this study. One of the potential applications of the nanofibres is textile yarn. Yarn is a fundamental element in textile products. Yarn made from nanofibres has high surface area and flexible. With the increase in the number of fibres in a unit area of cross section, the nanofibre yarn can improve some properties such as water and chemical absorbency as well as flexibility [13]. To form the yarn, the nanofibres should be able to be dyed because colour is one of the most important features in textiles particularly for military applications, smart apparel etc. [14].

**Figure 1:**
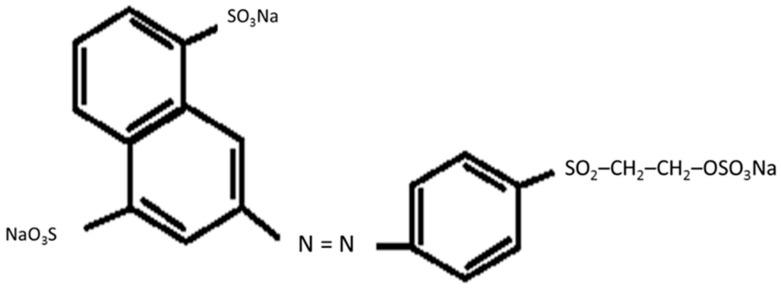
Chemical structure of Remazol Yellow FG.

There are several methods to dye electrospun nanofibres such as exhaustion, printing, padding and dope-dyeing [15,16]. Among these methods, dope-dyeing is more environmentally friendly as less chemicals and energy are needed due to facile processing and is requiring a direct addition of dye into the spinning solution [17]. In comparison to other methods, dope-dyeing promotes excellent dye dispersion, good colour uniformity, without any required chemical pre-treatment [18,19,20]. By adopting the dope-dyeing process, the negative impact of having residual dye waste, which later caused environmental pollution are avoidable [21,22]. A dye is known as coloured substance that has a chemical affinity to the underlying material to which it is being applied. There are many classes of dyes which are potentially used in dope-dyeing methods. Reactive dye is easy to bind to the textile by non-electrostatic forces, including hydrogen bonds, salt bridges, van der Waals forces and others [23,24], thus suitable to be applied in the electrospinning process [25]. Remazol Yellow FG is one of the common reactive dyes which contains the sulfato-ethyl-sulfone group (–SO_2_–CH_2_–CH_2_–OSO_3_Na) as their reactive group (Figure 1). According to Sultana (2016), the additional pre-treatment with alkali can cause the elimination of sulphuric acid to form a vinylsulfonyl moiety, which will allow it to react with cotton to form a covalent bond between dye and cotton fibre [26]. Another advantage of Remazol Yellow FG is easily soluble in water, very bright in colour, good in both wash fastness and light fastness and also has high application temperature, of up to 90 °C, which is compatible to be incorporated during the preparation of spinning solution [27,28].

Due to these outstanding features, the study has chosen Remazol to dye the electrospun nanofibers. Poly vinyl alcohol (PVA) is one of the synthetic polymers that compatible to be dyed with Remazol because the PVA has similar functional groups to cellulose [29]. Although the use of reactive dyes for electrospun fibres has been reported by other studies [16,30], but none have investigated the use of reactive dye in dope dyeing for electrospun fibres. In this work, a dope-dyeing of poly vinyl alcohol (PVA) nanofibres was carried out by colouring the polymer solution subjected to the electrospinning process. Dye in powder form was added to the pre-formed PVA solutions to fabricate dyed nanofibres by means of electrospinning process. To understand the formation of dyed PVA nanofibers using Remazol, the study has investigated the morphological structures and thermal decomposition behaviour of dyed fibres. In addition, the effect of dye content was carried out in order to identify colorimetric properties dyed PVA nanofibers.

## 2. Materials and Methods

In this research, the main polymeric material used to produce electrospun nanofibres was polyvinyl alcohol (PVA) in granules form with the mass average 125,000 g/mol (Mw) and 99% degree of hydrolysis (Sigma-Aldrich, St. Louis, MO, USA). Remazol Yellow FG dye and Ase Direct Supra Red BWS dye (in a powder form) were received from Aman Semesta Enterprise (Shah Alam, Selangor, Malaysia). All chemicals were used as received without further purification.

**Figure 2:**
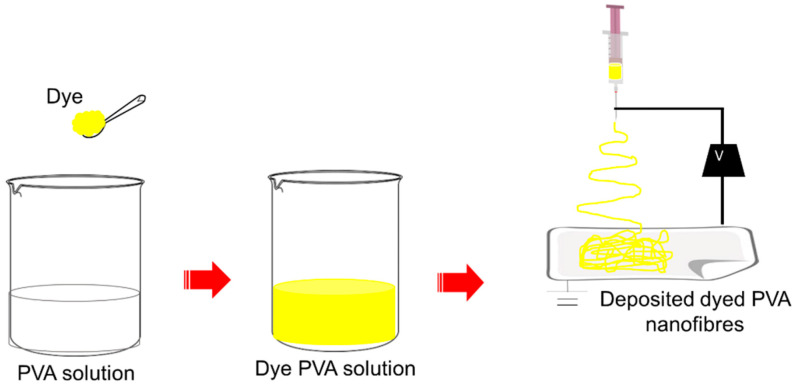
Illustration of dope-dyeing PVA nanofibres throughout electrospinning.

Two types of solutions were prepared in this study, which were PVA solution as control and dyed PVA solutions. For the control, 10 wt.% of PVA solution was prepared by dissolving the PVA granules in distilled water at 90 °C for 2 h. For the preparation of dyed PVA solutions, the Remazol Yellow FG dye was added in a different percentage. The dyed-polymer solution was first sonicated in an ultrasonic bath for 30 min and magnetically stirred until a clear solution was observed for 2 h at 90 °C. The dye-polymer relative percentage in weight used were 2.5, 5.0, 7.5, 10.0 and 12.5 wt.%, respectively. The resultant dye-polymer mixture solutions form a clear yellow solution and are well solubilized in the spinning solutions. A single nozzle spinneret with a 23G (internal diameter: 0.65 mm) needle was loaded with a spinning solution of dyed PVA at constant feed rate (0.5 mL/h) using a syringe pump. The electrospinning process was conducted at room temperature with relative humidity ranging from 40% to 60%. Solutions were subjected to be electrospin at the applied voltage of 15 kV with an electrospinning distance of 10 cm for 5 min to collect the deposited nanofibres. Figure 2 illustrates the dope-dyeing process of PVA nanofibres via electrospinning throughout this study. The preparation of Ase Direct Supra Red BWS solution was similar to Remazol Yellow FG with dye concentrations of 0.2 wt.%, 0.4 wt.%, 0.6 wt.%, 0.8 wt.% and 1.0 wt.%, respectively. The solution was electrospun at similar conditions to Remazol Yellow FG.

The surface morphology of resultant electrospun PVA nanofibres were characterized using Hitachi model TM3030 Plus, Hitachi High Technologies America, Inc. Scanning Electron Microscopy (SEM) (Schaumburg, IL, USA). The chemical structure changes in the electrospun nanofibres incorporated with dye were determined using Fourier Transform Infra-Red Attenuated Total Reflection Spectroscopy (FTIR ATR) analysis. The absorbance measurements were performed using the Perkin Elmer model 1000 series. FTIR spectra were recorded in the wavenumber range from 4000 to 400 cm^−1^ and were signal averaged from a minimum of 10 scans per sample to reduce noise. The colour hue of the dyed electrospun nanofibres were analysed using colour space colorimeter CIE L* a* b* (CIELAB) (Hunter Labscan XE Colour Measurement, Reston, VA, USA)In this study, the optimal dye content was determined according to clear coloured fibres formation without impurities such as droplets and splashes. Thermal decomposition behaviour of dyed PVA nanofibres was characterized using thermogravimetric (TGA) analysis (Setaram Setsys Evolution instrument, Lyon, France) at a heating rate of 10 °C/min in the temperature range between 50 to 600 °C. The scans were performed under a nitrogen atmosphere. The determination of weight loss versus temperature was analyzed from the TGA curve.

In order to improve the hydrophilic properties of the dyed electrospun nanofibres, the prepared nanofibres samples were cross-linked by placing them under a vapour of glutaraldehyde in a desiccator for 24 h at room temperature. After 24 h, the nanofibres samples were removed from the desiccator and were dried at room temperature overnight to remove the excess glutaraldehyde. For UV-Visible spectroscopy analysis, the cross-linked nanofibres samples were incubated for 24 h in distilled water and the absorption spectrum of the supernatants were recorded using UV-Vis spectrometer (Perkin Elmer, Lambda 35, Norwalk, CT, USA) from 800 to 200 nm. 10 µg/mL of Remazol Yellow FG solution was prepared as a control sample.

Fluorescence emission spectra of dyed electrospun nanofibres solid samples were recorded with fluorescence spectrometer (Perkin Elmer, LS 55, Cambridge, MA, USA) when induced by UV-light between 310–400 nm. All the emission spectra were recorded with an excitation slit width of 5 nm.

## 3. Results and Discussion

### 3.1. Effect of Dye Concentration on Fibre Diameter

**Figure 3:**
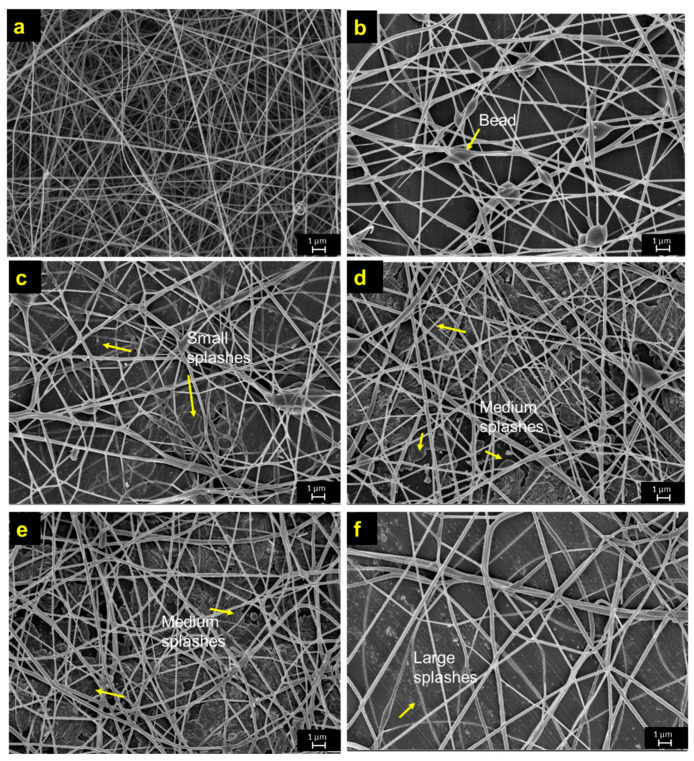
SEM micrographs of (**a**) control PVA (**b**) 2.5 wt.% (**c**) 5.0 wt.% (**d**) 7.5 wt.% (**e**) 10.0 wt.% (**f**) 12.5 wt.% of dyed PVA nanofibres using Remazol FG yellow dye.

Figure 3a depicted the morphological structure of control PVA nanofibres in a long, continuous and uniform fibrous shape. The mean fibre diameter of the control PVA nanofibres was approximately 138.1 ± 12 nm. Figure 3b–f depicted the changes on fibre diameter by varying the concentration of dye. It was observed that there was a slight increment of fibre diameter with the formation of bead-on-string structures as dye was introduced at the concentration of 2.5 wt.% (Figure 3b). The mean fibre diameter of the dyed PVA nanofibres at 2.5 wt.% was approximately 141.7 ± 12.7 nm. The probable reason for beaded fibres formation was due to the insufficient elasticity of the dye-polymer solution mixture upon the elongation period during the electrospinning process. The formation of beaded fibres was also observed by other studies [31,32,33]. The authors explained the formation of beaded fibres was attributed to an insufficient stretch of the filaments during the whipping of jet [32]. A gradual increase in fibres diameter was revealed along with the increment of dye concentrations as expected (Figure 3c–f). The mean fibre diameter of dyed PVA nanofibres was 160.8 ± 13.3 nm, 176.5 ± 10.8 nm, 177.1 ± 11.5 nm and 186.4 ± 13.2 nm with dye content of 5.0 wt.%, 7.5 wt.%, 10.0 wt.%, and 12.5 wt.%, respectively. The enlargement in the fibre diameter was in correlation to the dominant effect from the increment of the dye-polymer solution viscosity in this study [34]. The formation of smooth, long, continuous dyed nanofibres were observed as the range of dye content was increased from 5.0 wt.% to 12.5 wt.% as in Figure 3c–e.

**Figure 4:**
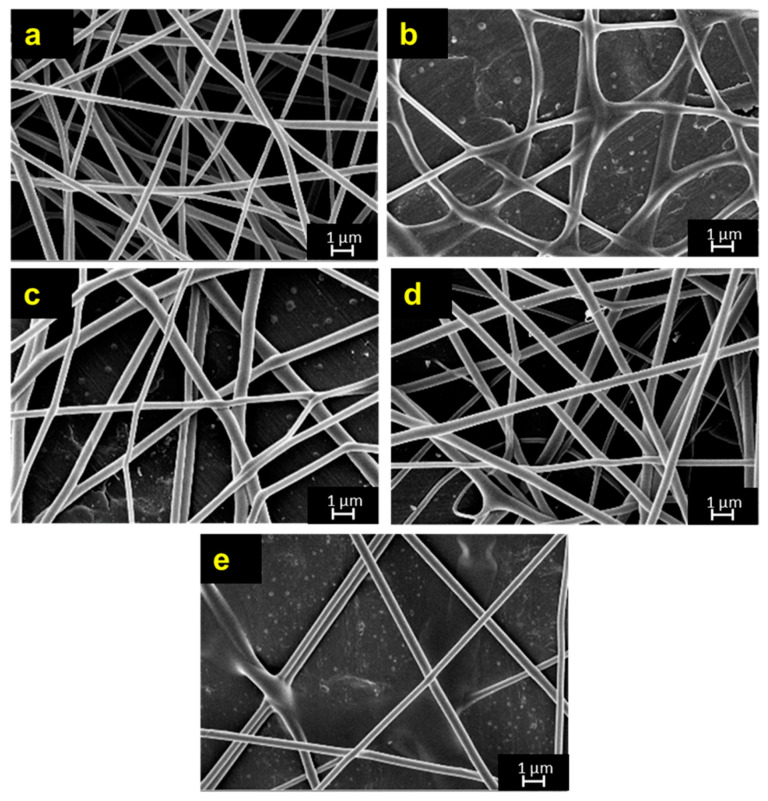
SEM micrographs of (**a**) 0.2 wt.% (**b**) 0.4 wt.% (**c**) 0.6 wt.% (**d**) 0.8 wt.% (**e**) 1.0 wt.% of dyed PVA nanofibers using Ase Direct Supra Red BWS dye.

Aside from Remazol Yellow FG, the study has conducted another preliminary work on the use of direct dye (Ase Direct Supra Red BWS) in the colouration of electrospun PVA nanofibres. The changes of fibre diameter using Ase Direct Supra Red BWS dye are illustrated in Figure 4a–e. As expected, the resultant fibre diameter increases from 214.72 ± 45 nm to 395.00 ± 82 nm as the amount of dye increases from 0.2 wt.% to 1.0 wt.%. At higher dye concentrations, the solution viscosity is expected to increase, resulting the formation of large fibre diameter. Similar findings on the effect of dye concentration on fibre diameter were also observed by other studies [7,34]. In addition, the Ase Direct Supra Red BWS dye forms a larger fibre diameter than the Remazol Yellow FG. This could be due to the large molecular size of direct dye used in the study.

### 3.2. Effect of Dye Concentration on Fibre Morphology and Colorimetric Properties of Dyed Fibres

The dye concentration has a certain influence on the fibre morphology as well as the colorimetric properties of dyed PVA nanofibres. The macroscopic droplets were observed in the SEM micrographs of dyed PVA nanofibres with the usage of 12.5 wt.% dye content as depicted in Figure 3f. The current obtained results are in accordance with another finding by Fantini and Costa (2009), which reported that the macroscopic dropping phenomenon started as the stability of Taylor cone distorted at the higher percentage of dye consumption [12]. It was noticed that the presence of droplets among the deposited fibres would reduce the quality of fibre morphology. The formation of fibre impurities (i.e., splash, droplet) within nanofibre mesh during electrospinning process would consequently lead to gradual deterioration of fibre morphologies [35,36,37].

The colorimetric coordinates of L* a* b* for dyed PVA nanofibres were reported in Table 1. The coordinate L* defines a lightness axis, a* a redness–greenness axis, and b* a yellowness–blueness axis. The positive value of b* colorimetric coordinates indicated the yellowness of dyed PVA nanofibres in this study. The yellowness (demonstrated by an increase in b* values) of each sample increased with an increase in dye content. As the dye content is increased to 12.5 wt.%, the value of b* colorimetric coordinates decreased as affected from the poor fibres formation. On the other hand, the shape of the nanofibres deposition spot varied depending on the jet stability during the electrospinning process. The membrane coverage areas of dyed PVA nanofibres were increased upon the increment of dye content, as a result of rapid projection of ejected fibre jet which was controlled by the solution viscosity [38,39].

The morphological analysis of dyed PVA nanofibers using Ase Direct Supra Red BWS is illustrated Figure 4. Cylindrical fibres were observed at each concentration, suggesting that the fibres had sufficient polymer chain entanglement at each concentration. The formation of red dyed PVA nanofibres was clearly seen on the collector. These dyed fibres were further tested for colorimetric analysis. As tabulated in Table 2, the 0.8 wt.% of dyed PVA exhibits the highest redness (a* value) as compared to other dye concentration. The result also shows that the increase of dye content does not increase the redness values, which is contradicted to previous study [18]. This could be due to the use of different colourants for the formation of dyed fibres. Previous study used pigment to form dyed fibres, whereas the current study used dyes to form the dyed fibres.

### 3.3. FTIR ATR Analysis

**Figure 5:**
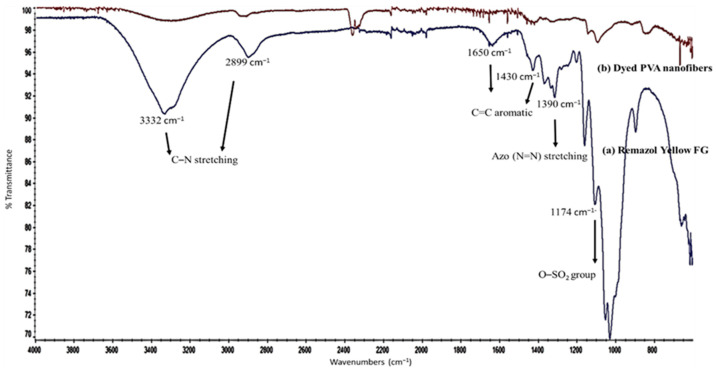
FTIR ATR spectra of (**a**) Remazol Yellow FG dye and (**b**) dyed PVA nanofibres.

The presence of Remazol Yellow FG dye in the dyed PVA nanofibres was determined using FTIR ATR analysis by the association to the Remazol Yellow FG IR spectrum. From the IR spectrum of Remazol Yellow FG shown in Figure 5a, the following peaks at 3332 cm^−1^ and 2899 cm^−1^ were assigned to the C–N stretching band, where the peaks at 1650 cm^−1^ and 1430 cm^−1^ were assigned to the C=C aromatic stretching band. The prominent peak of azo group (N=N) stretching band in Remazol Yellow FG was clearly shown at 1390 cm^−1^ while the sulfonic salt functional group was observed at 1174 cm^−1^. From the literatures, the hydroxyl region in IR spectrum in PVA is typically split into sub-regions depending on the type of hydrogen bonding interaction occurring [40]. More specifically, the broad band observed between 3550 cm^−1^ and 3200 cm^−1^ is associated with the O–H stretching band from the intermolecular and intramolecular hydrogen bonds [41]. The peak assigned for these free hydroxyl belongs to OH group in the polymer is diminishes as shown in IR spectrum of dyed PVA nanofibres according to Figure 5b). Technically, it is suggested the diminished of the particular peak is due to the occupied OH group bonded to dye molecules. Throughout the analysis, it had been confirmed that the presence of all prominent peaks of Remazol Yellow FG in the sample of dyed PVA nanofibres as shown in the IR spectrum of dyed PVA nanofibres in a reduced peak intensity (Figure 5b).

### 3.4. TGA Analysis

**Figure 6:**
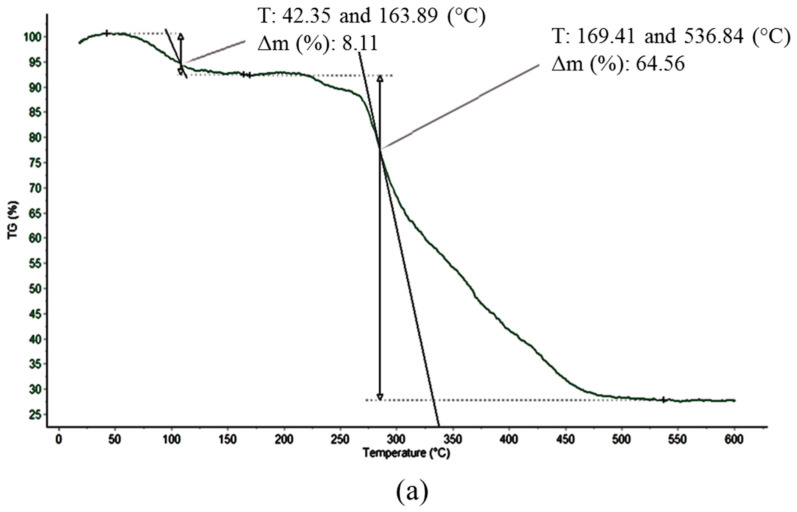

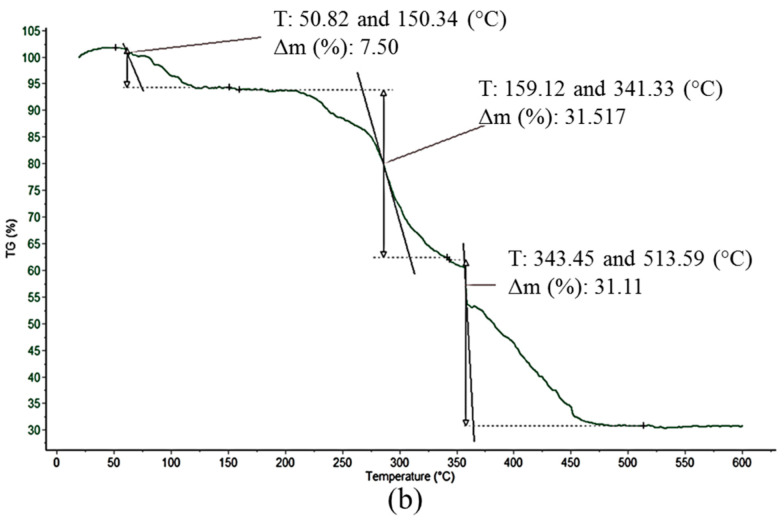
TGA thermogram of (**a**) PVA (**b**) dyed PVA electrospun nanofibres.

The thermal stability of PVA nanofibres upon the embedment of dye was analyzed using TGA analysis by monitoring weight losses under inert atmosphere. Figure 6a,b show the weight loss as a function of temperature of both electrospun PVA and dyed PVA nanofibres. For electrospun PVA nanofibres, a small weight loss of 8.11% was observed at the temperature range of 42.35 to 163.89 °C, indicating the presence of free moisture in the sample. Whereas, 64.56% of PVA decomposition was observed at the temperature range of 169.41 to 536.84 °C. Meanwhile, the TGA thermogram of dyed PVA nanofibres shows three weight loss steps in which represents a discrete decomposition events at a given temperature. A small weight loss was observed at the temperature range of 50.82 to 150.34 °C, which was attributed to the evaporation of moisture. The second degradation step at the temperature range of 159.12 to 341.33 °C was ascribed to the decomposition of PVA. Whereas, a weight loss of 31.11% occurred at the temperature of 343.45 to 513.59 °C is an indication of the degradation of the polymer phase reinforced with dye. The addition of dye into electrospun PVA nanofibres has resulted in the increment of thermal stability of PVA due to the reduced chain mobility of dye mixed with polymer matrix.

### 3.5. UV Visible Spectroscopy Analysis

**Figure 7:**
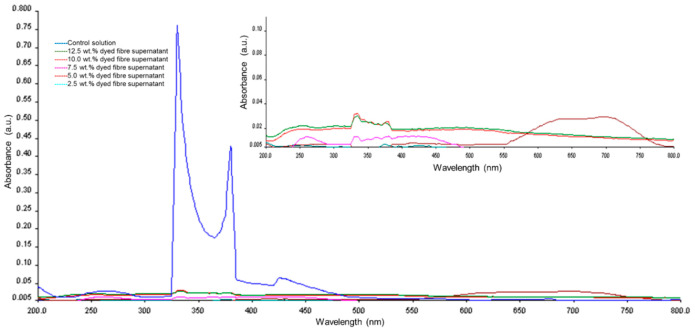
UV-Visible spectra of Remazol Yellow FG solution and in vitro release of Remazol Yellow FG supernatant originated from dyed electrospun nanofibres. The inset shows the zoom-in UV-Visible spectra of dyed electrospun nanofibres.

The spectrophotometric detection of Remazol Yellow FG in vitro release was carried out by using UV-Visible spectroscopy. The strong absorbance peaks were recorded at UV range of 260 nm, 330 nm and 380 nm and visible range of 440 nm for control Remazol Yellow FG solution. For the detection of dye in vitro release, the prominent characteristic peaks for the Remazol Yellow FG at 260 nm, 330 nm, 380 nm and 440 nm were significantly decreased for all dyed electrospun fibres supernatants. The data from UV-Vis absorption spectrum has shown low absorbance at λ_max_ 330 nm, showing minimal sign of dye release, thus suggesting a good dye blending in the polymeric matrix of dyed electrospun nanofibres. In this study, we concluded that the cross-linked dyed electrospun fibres have the ability to keep their structures intact in aqueous. The reduction of the polymer hydrophilicity is originated from the bonding of polymer network chains to glutaraldehyde together during the cross-linking process which prevents water from entering into the nanofibres network [42]. Figure 7 shows the UV-Visible spectra of control Remazol Yellow FG solution and in vitro release of Remazol Yellow FG supernatants originating from dyed electrospun nanofibres sample, respectively.

### 3.6. Flourescent Spectroscopy Analysis

**Figure 8:**
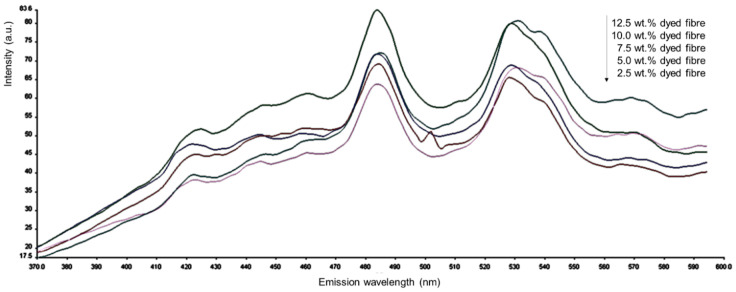
Fluorescence emission spectra of Remazol Yellow FG dyed PVA nanofibres.

The fluorescence spectra of all dyed electrospun nanofibres with Remazol Yellow FG have shown that their fluorescence spectra overlap heavily with each other. As shown in Figure 8, the Remazol Yellow FG can emit fluorescence due to the presence of some fluorescent chromophore and auxochrome groups existing in the molecules, such as =C=O, >C=N–, benzene ring, naphthalene ring, –OH, –SO_3_Na. They are connected together through azo bond, leading to the formation of conjugated double-bond systems. Electrons existing in it can easily absorb photons by the transition form of π → π* and emit fluorescence. In addition, the conjugacy of molecules is enhanced when the substituents –SO_3_Na and –SO_3_Na place at the para position of naphthalene ring, and the molecules associate together through hydrogen bond [43,44]. Figure 8 and Table 3 shows the fluorescence emission spectra and spectrum characteristic of Remazol Yellow FG, respectively.

### 3.7. Dye-Polymer Interaction

**Figure 9:**
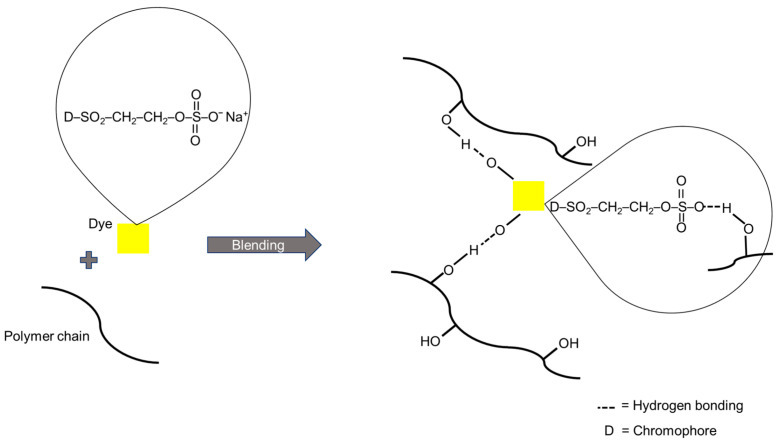
Schematic illustration of the proposed mechanism of dye-polymer interaction.

A schematic illustration of the likely mechanism of the chemical interaction between dye solute and polymer chains is shown in Figure 9. In this study, the possible interaction between the dye solute and the polymer network is based on the intermolecular hydrogen bonding [23]. The sulfato-ethyl-sulfone group (–SO_2_–CH_2_–CH_2_–OSO_3_Na) of the dye solute is being bonded to the hydroxyl functional groups of the polymer chains. It is elucidated that both of the dye-polymer intermolecular interactions and dye-dye self-association interactions naturally occur in the blend solution mixture. The relative magnitudes and strengths of these interactions however depend on the types of functional group present in the polymer matrix and the concentration of the dye solute in the polymer blend [45]. The addition of dye has initially interrupted intra and intermolecular hydrogen bonding in the PVA network. The increasing of the concentration of dye solute in this works has altered the viscosity of the polymer blend. It is suggested the presence of dye solute has induced the intermolecular bonding between dye-polymer, reduced the polymer chains mobility thus increased the entanglement of the polymer blend network [46]. The increment of the viscosity of the dye-polymer blend has led to a reduction of the polymer elongation, resulting in a bigger fibre diameter during the electrospinning process.

## 4. Conclusions

The preliminary study on dope-dyeing of PVA nanofibres using Remazol Yellow FG through electrospinning process was successful, clearly observed in shades of yellow. Characterization by SEM analysis confirmed the enlargement in the fibre diameter of dyed PVA nanofibres upon the increment of dye content from 2.5 wt.% up to 12.5 wt.%. Further increase in dye content was found to affect whipping instabilities in electrified liquid jet during the electrospinning, resulting in the macroscopic dropping phenomenon. The experimental works herein concluded that the optimal Remazol Yellow FG content for the colouration of PVA nanofibres is at 10.0 wt.% due to the high value of colour hue and good fibres morphology, without any fibre impurities. In addition, the presence of all prominent peaks of Remazol Yellow FG in the PVA nanofibers was confirmed by the FTIR analysis. From the TGA analysis, the addition of dye into electrospun PVA nanofibres has resulted in the increment of thermal stability of the PVA due to the reduced chain mobility of dye mixed with polymer matrix. The detection of Remazol Yellow FG in-vitro release and fluorescence properties of Remazol Yellow FG dyed electrospun nanofibres can be recognized accurately by UV-visible spectroscopy and fluorescence spectroscopy analysis, respectively. Further studies on the effect of lower concentration of dye on fibres, colour fastness to light, wash and rub properties shall be conducted to evaluate the dyeing efficiency of electrospun nanofibres by means of electrospinning process.

## Figures and Tables

**Table 1:** The colorimetric coordinates of L* a* b* for dyed PVA nanofibers Remazol FG yellow dye.

Dye Content (wt.%)	L*	a*	b*
2.5	6.05	1.23	10.28
5.0	6.39	3.74	10.78
7.5	5.02	5.21	8.62
10.0	11.68	9.85	20.03
12.5	9.79	7.98	16.77

**Table 2:** The colorimetric coordinates of L* a* b* for dyed PVA nanofibers using Ase Direct Supra Red BWS dye.

Dye Content (wt.%)	L*	a*	b*
0.2	0.37	1.54	0.64
0.4	0.31	1.32	0.54
0.6	0.28	1.25	0.48
0.8	0.19	1.04	0.33
1.0	0.03	0.18	0.05

**Table 3:** Fluorescence spectrum characteristic of Remazol Yellow FG.

Sample	Remazol Yellow FG
Max Emission wavelength (nm)	530
Fluorescence peak (nm)	484, 530
Fluorescence spectra width (nm)	450–600
